# A Reappraisal of Thymosin Alpha1 in Cancer Therapy

**DOI:** 10.3389/fonc.2019.00873

**Published:** 2019-09-06

**Authors:** Claudio Costantini, Marina M. Bellet, Marilena Pariano, Giorgia Renga, Claudia Stincardini, Allan L. Goldstein, Enrico Garaci, Luigina Romani

**Affiliations:** ^1^Department of Experimental Medicine, University of Perugia, Perugia, Italy; ^2^Department of Biochemistry and Molecular Medicine, School of Medicine and Health Sciences, The George Washington University, Washington, DC, United States; ^3^University San Raffaele and IRCCS San Raffaele, Rome, Italy

**Keywords:** thymosin alpha1, checkpoint inhibitors, immunotherapy, colitis, dendritic cells

## Abstract

Thymosin alpha1 (Tα1), an endogenous peptide first isolated from the thymic tissue in the mid-sixties, has gained considerable attention for its immunostimulatory activity that led to its application to diverse pathological conditions, including cancer. Studies in animal models and human patients have shown promising results in different types of malignancies, especially when Tα1 was used in combination with other chemo- and immune therapies. For this reason, the advancements in our knowledge on the adjuvant role of Tα1 have moved in parallel with the development of novel cancer therapies in a way that Tα1 was integrated to changing paradigms and protocols, and tested for increased efficacy and safety. Cancer immunotherapy has recently experienced a tremendous boost following the development and clinical application of immune checkpoint inhibitors. By unleashing the full potential of the adaptive immune response, checkpoint inhibitors were expected to be very effective against tumors, but it soon became clear that a widespread and successful application was not straightforward and shortcomings in efficacy and safety clearly emerged. This scenario led to the development of novel concepts in immunotherapy and the design of combination protocols to overcome these limitations, thus opening up novel opportunities for Tα1 application. Herein, we summarize in a historical perspective the use of Tα1 in cancer, with particular reference to melanoma, hepatocellular carcinoma and lung cancer. We will discuss the current limitations of checkpoint inhibitors in clinical practice and the mechanisms at the basis of a potential application of Tα1 in combination protocols.

## Thymosin α1: The Origin

The origin of thymosin dates back to 1961 when it was shown that neonatal thymectomy had severe consequences on the immunological capacity of newborn animals (1). These effects included a marked deficiency of lymphocyte populations of the blood and the lymphoid tissue, inability to elicit cell-mediated immune responses and produce antibodies in response to the administration of some antigens, and the development of a syndrome described as wasting disease, characterized by a failure to grow at a normal rate with atrophy of the lymphoid tissue (1). Such effects could be prevented by thymic grafts, which were still effective when enclosed in cell-impermeable chambers, thus leading to major efforts to isolate and characterize soluble, biologically active, thymic factors (1–3). Goldstein et al. first reported the preparation and partial purification of a thymic factor, termed thymosin, that was able to induce lymphocytopoiesis in CBA mice (4), prevent wasting disease (5), and restore immunological competence in thymectomized mice, as assayed by the graft-vs-host (6) and skin allograft responses (7). This thymosin preparation, termed thymosin fraction 3, was further purified to yield a highly active preparation, thymosin fraction 5, that could be prepared in large amounts and was suitable for clinical use (8). Thymosin fraction 5 contains at least 40 polypeptides, with molecular weight ranging from 1,000 to 15,000 Da, and isoelectric points at pH 3.5–9.5, based on which individual peptides are designated as α (isoelectric points below 5), β (5.0–7.0), and γ (above 7.0). The first peptide isolated from the thymic tissue was in the α region, and termed thymosin alpha 1 (Tα1) (9), characterized as an N-terminal acetylated acidic peptide of 28 amino acids with a molecular weight of 3,108 Da, active in *in vitro* and *in vivo* assays of T cell differentiation and function (9).

## Thymosin α1 and Cancer: The Rationale and the Early Clinical Studies

Based on the immunostimulatory activities of thymosin, early clinical trials assessed the efficacy of thymosin fraction 5 and Tα1 in patients with primary immunodeficiencies as well as in cancer patients (2). The rationale for the use of thymosin in cancer patients would be to enhance the immune capabilities with two aims: combating the tumor more efficiently and preventing opportunistic infections. In addition, the use of thymosin would counteract the immunosuppressive side effects associated with conventional chemotherapy and radiotherapy (10). The first phase I clinical trial with Tα1 was performed at the National Cancer Institute (11) while subsequent clinical trials were endorsed by the Biological Response Modifier Program (BRMP), within the Division of Cancer Treatment, with the responsibility to foster the development of biologicals, including thymosin fraction V and Tα1, that have therapeutic efficacy in cancer (12). As a result, BRMP-sponsored studies indicated evidence of both clinical antitumor response and biological modification in patients with relatively small tumor burden and receiving local radiation therapy, while little clinical or biological activity was noted in patients with advanced disease (10, 12). The administration of thymosin was associated with a favorable safety profile (10). Pre-clinical screening did not show enhancement of Natural Killer (NK) cell and macrophage tumoricidal activity, but increase of T cell responses following *in vitro* and *in vivo* stimulation, including antitumor efficacy in B16 melanoma-based models of experimental and spontaneous metastasis (12).

## Thymosin α1 and Cancer: From the Early Clinical Studies to the Current Status

Several studies on a variety of tumors have been performed to assess the safety and efficacy of Tα1 in cancer therapy from the early clinical studies to the more recent years. In the following sections, we will present and discuss the studies performed in murine models and human patients of melanoma, hepatocellular carcinoma and non-small cell lung cancer, for which more evidence has been accumulated (Table 1).

**Table 1:** Pre-clinical and clinical studies with Tα1 in cancer and chronic hepatitis B and C.

**Pathology**	**Study**	**Treatment**	**Efficacy**	**References**
Melanoma	Pre-clinical	Tα1 monotherapy	Evidence of treatment efficacy	(15, 30)
			No significant evidence of treatment efficacy	(17–19, 31, 33–35)
		Tα1 in combination therapy with: αβ-IFN IL-2 Cy and αβ-IFN anti-PD-1 Ab	Evidence of treatment efficacy Evidence of treatment efficacy Evidence of treatment efficacy Evidence of treatment efficacy	(17–19) (19) (20) (30)
		Tα1 fusion proteins: - concatemer - thymopentin - RGDR - iRGD - Fc	Evidence of treatment efficacy Evidence of treatment efficacy Evidence of treatment efficacy Evidence of treatment efficacy Evidence of treatment efficacy	(31) (32) (33) (34) (35)
	Clinical	Tα1 in combination therapy with: - dacarbazine and IL-2 - dacarbazine and IFNα - dacarbazine and IFNα - dacarbazine	Evidence of treatment efficacy Evidence of treatment efficacy Evidence of treatment efficacy Evidence of treatment efficacy	(25) (26) (27, 29) (28, 29)
Chronic hepatitis B	Clinical	Tα1 monotherapy	Evidence of treatment efficacy	(40, 42–44)
			No evidence of treatment efficacy	(46, 47)
		Tα1 in combination therapy with: - IFNα - pegylated IFNα - famciclovir - lamivudine - entecavir	Evidence of treatment efficacy No evidence of treatment efficacy Evidence of treatment efficacy Evidence of treatment efficacy Evidence of treatment efficacy No evidence in HBV-related compensated cirrhosis	(48–51) (52) (62) (63) (64) (65)
Chronic hepatitis C	Clinical	Tα1 monotherapy	No evidence of treatment efficacy	(54, 55)
		Tα1 in combination therapy with: - IFNα - IFN and ribavirin	Evidence of treatment efficacy Evidence of treatment efficacy No evidence in on-treatment viral response	(53, 56–58) (66–69) (70)
HCC	Clinical	Tα1 monotherapy	Evidence of treatment efficacy	(71, 72)
		Tα1 in combination therapy with: - TACE - lamivudine - sorafenib	Evidence of treatment efficacy Evidence of treatment efficacy Evidence of treatment efficacy	(73–75) (76) (77)
Lung cancer	Pre-clinical	Tα1 monotherapy	Evidence of treatment efficacy	(19, 85–87, 89)
			No significant evidence of treatment efficacy	(17, 24, 34, 80, 84)
		Tα1 in combination therapy with: - αβ-IFN - Cy and αβ-IFN - Cy and IL-2 - gemcitabine	Evidence of treatment efficacy Evidence of treatment efficacy Evidence of treatment efficacy Evidence of treatment efficacy	(17, 19) (80) (24) (84)
		Tα1 fusion protein: - iRGD	Evidence of treatment efficacy	(34)
	Clinical	Tα1 monotherapy	Evidence of treatment efficacy	(79)
		Tα1 in combination therapy with: - cisplatin, etoposide, IFNα2a - ifosfamide, IFNα - cisplatin, vinorelbine or gemcitabine	Evidence of treatment efficacy Trend toward treatment efficacy Evidence of treatment efficacy	(81) (82) (83)

## Thymosin α1 and Melanoma

The first observations that Tα1 could play a protective role in melanoma came from the work of Ishitsuka et al. who had previously shown that Tα1 was able to protect 5-flurouracil immunosuppressed mice from infection by opportunistic pathogens (13). The authors asked whether Tα1 could similarly protect mice immunosuppressed with cytostatics or X-ray irradiation, and inoculated with B16 melanoma or leukemic cells, from metastatic growth (14, 15). As a result, Tα1 was found to increase survival and reduce the incidence of metastasis by preventing the reduction of NK cell activity and preserving the barrier integrity from tumor cell spreading (14, 15). Along the same line, and moving from their previous observation that Tα1 and αβ-IFN could stimulate NK cell activity in cyclophosphamide (Cy)-immunosuppressed mice (16), Pica and coauthors demonstrated that mice inoculated with B16 melanoma or Lewis lung carcinoma (LLC) cells had restored NK cell activity if treated with Tα1 and αβ-IFN 10 days after tumor inoculation (17, 18). Since the single treatments were ineffective, the results of the two studies were interpreted as lack of mature NK cells upon immune impairment by chemotherapy or tumor growth, with Tα1 promoting the maturation of progenitors that became responsive to αβ-IFN stimulation with increased cytolytic potential (16–19). The anti-tumor effects were improved in B16 melanoma-bearing mice by the combined chemo-immunotherapy with Cy, high doses of Tα1 and low dose αβ-IFNs (20).

A similar ability of Tα1 to induce the maturation of progenitors into mature cytolytic cells has been also hypothesized in a study using the combined treatment with Tα1 and IL-2 (19, 21), a result in line with the ability of Tα1 to induce IL-2 receptor expression (22, 23). Collectively, these studies suggest that pre-treatment with Tα1 could restore the boosting capacity of both αβ-IFN and IL-2 with a favorable safety profile and may be used as adjuvant in cancer therapy.

Based on these observations, and prompted by a study showing that the combined treatment with Cy, Tα1, and IL-2 was superior to the single agents, or the combination of Cy with either Tα1 or IL-2, in improving survival of mice with LLC (24), a phase II study with metastatic melanoma patients treated with dacarbazine, Tα1 and IL-2 was performed (25). Although the absence of a group treated only with dacarbazine prevented comparison between the different regimens, the results were promising with objective responses observed in 36% of patients and no particular safety concerns, with no overlapping toxicity and interference between the different agents (25). A second phase II open label trial was performed by the same group to evaluate the efficacy of dacarbazine, Tα1 and IFNα in advanced metastatic melanoma patients (26). Objective response was observed in 50% of patients with no additional toxicities. Moreover, immune evaluation in twelve patients revealed beneficial effects on NK cell activity and CD4+ cell number after the suppression induced by dacarbazine when compared to matched melanoma patients treated with dacarbazine alone.

Based on these results and the reinforced notion that the combination of immunotherapy and chemotherapy may be beneficial in melanoma because of its immunogenicity, a phase II, multicenter, open, randomized, dose ranging study was performed to investigate the safety and efficacy of different doses of Tα1 in combination with dacarbazine and with or without IFNα in stage IV melanoma (27). This study confirmed that administration of Tα1 did not result in additional toxicity while increasing the efficacy of the treatment as evident from the higher clinical benefit rate and a trend toward improved OS and higher PFS with any Tα1-containing regimen (27). While the mechanism(s) at the basis of these effects are unknown, but likely involving the immunomodulatory activities of Tα1, this study further encourages the use of Tα1, in combination with chemotherapy, in the treatment of metastatic melanoma. This concept was substantiated by a Tα1 compassionate use program in which thirty-one patients with advanced-stage malignant melanoma were treated with Tα1 and dacarbazine and a clinical benefit rate of 41% was observed (28). Interestingly, the patients enrolled in the two studies were further analyzed in a long-term follow-up study and an encouraging 13.3 months median OS was observed, with indications that a proportion of patients benefits for a long time from the treatment with Tα1 (29). The study also analyzed possible interactions with immune checkpoint blockade antibodies. Of note, when the analysis was focused on patients that received ipilimumab in a second or subsequent line of therapy, the median OS was 38.4 months if Tα1 was administered before ipilimumab, compared to 8 months with ipilimumab alone, irrespective of timing from last Tα1 treatment, Tα1 dosage or Tα1 cycles. These results point to a synergistic effect of a sequential Tα1 and ipilimumab regimen, and, as speculated by authors, to an immune-maintenance role of Tα1 in addition to the well-known immune priming activity (29).

The pre-clinical and clinical studies described above demonstrate a potential role of Tα1 as adjuvant in melanoma therapy, but whether Tα1 could also play an anti-tumoral activity as monotherapy has remained unclear. A recent evaluation of Tα1 in a mouse melanoma lung metastasis model has shown for the first time that Tα1 can significantly decrease lung metastasis as monotherapy and, at lower doses, while being ineffective alone, reduced metastasis when combined with an anti-PD-1 antibody (30). In addition, Tα1 was also effective as monotherapy in a syngeneic model of melanoma tumor growth using the highly metastatic B16.F10 clone (30). The discrepancy with previous reports on the inefficacy with Tα1 as monotherapy might be linked to differences in the administration protocols, which include timing, route, and dosage. Given the pleiotropic activities of Tα1, it is likely that the different functions are tailored to the levels and the temporal and spatial variations of active Tα1 such that the modulation of the tumor and/or the environment at local and distant sites are variably affected. In support of such hypothesis are a series of papers in which fusion proteins with Tα1 are synthesized and the different physicochemical properties account for distinct functions of the molecule in melanoma. For instance, a Tα1 concatemer induced apoptosis of B16 cells more effectively than Tα1, and reduced tumor growth and weight in B16 melanoma bearing mice while Tα1 was ineffective (31). Similarly, a Tα1–thymopentin fusion peptide, in combination with Cy, reduced tumor weight more efficiently than Cy and Tα1, with or without thymopentin (32). Finally, the addition of an RGDR or iRGD motif at the C-terminus to favor tumor homing and cell internalization, or the Fc domain of human IgG4, that considerably increased the half-life of Tα1, resulted in higher anti-tumor effects compared to Tα1, with higher levels of IFNγ and IL-2 and higher lymphocyte infiltration (33–35). All in all, pre-clinical and clinical experience with Tα1 in melanoma suggest that at least three possible mechanisms may be brought into play to explain the beneficial activity of Tα1: first, a direct effect on tumor cells; second, an immune priming for the activity of chemo- and immunotherapies; third, immune maintenance for long-term protection, each function likely favored by the pattern of bioactive Tα1, immune and tumor status, and concomitant or previous therapies.

## Thymosin α1 and Hepatocellular Carcinoma

### Thymosin α1 as a Prevention of HCC in Chronic Hepatitis B and C

Hepatocellular carcinoma (HCC) is the most common type of primary liver cancer in adults and about three-quarters of HCCs are attributed to chronic HBV and HCV infections (36). Chronic hepatitis B is the most frequent etiology of HCC in countries with scarce medical resources while hepatitis C and alcoholic liver disease are most common risk factor for HCC in the Western countries (36). Moving from observations on the effects of thymosin fraction 5 on T lymphocytes in alcoholic liver disease (37) and chronic active hepatitis (38, 39), Mutchnick et al. performed a pilot clinical study in patients with chronic hepatitis B to assess the safety and efficacy of thymosin fraction 5 and Tα1 (40). Although the quality of the study has been questioned (41), indications were suggestive of a beneficial effect of thymosin by promoting disease remission and cessation of virus replication in the absence of side effects, with higher lymphocytes count and increased IFNγ production (40). Long-term follow-up of the responder patients (2–5 years) demonstrated a sustained response to treatment, and an additional study confirmed a response to therapy in six of seven patients with anti-HBe(+) chronic hepatitis B (42). Similarly, Tα1 proved effective in the subpopulation of chronic hepatitis B patients that lack HBeAg when compared to IFNα, with the advantage to be well-tolerated (43), and arrested HBV replication and reduced lobular activity in 40% of patients in a subsequent randomized, controlled trial (44). The action of thymosin was more likely related to the restoration of the immune competence to eliminate HBV and resolve the inflammatory process, rather than to a direct antiviral activity, with a delayed effect (43–45).

However, a multicenter, randomized, double blind and placebo-controlled study failed to confirm these observations (46). Indeed, while there was a trend toward efficacy with Tα1, it did not reach statistical significance (46). Similarly, a randomized controlled trial in the subpopulation of patients infected with pre-core mutants as in Andreone et al. (43), could not identify increased response rates with Tα1, although a reduction of immune-mediated liver cell necrosis was likely present (47), thus raising doubts regarding the possible use of Tα1 as monotherapy in chronic hepatitis B.

At the same time, however, the notion that combining an antiviral agent with an immunomodulatory molecule could improve the response rate was emerging, thus paving the way for clinical trials testing the use of Tα1 in combination with IFN. A first study was performed to assess the safety and efficacy of low dose lymphoblastoid IFNα and Tα1 in patients with chronic hepatitis B, either naïve or non-responsive to standard IFN-α2b (48). A response was observed in 60% of patients with no reactivation of the disease beyond the 12-month follow-up period, and adverse events associated with IFNα were mild (48), a result confirmed in a subsequent study (49), while a randomized, placebo-controlled trial revealed a trend toward increased response rates with IFNα and Tα1 vs. IFNα alone (50). The combination of Tα1 and IFNα-2b also proved effective in HBeAg negative chronic hepatitis B patients as compared to IFNα-2b monotherapy or in combination with lamivudine (51). On the contrary, however, Tα1 and pegylated IFNα-2a did not prove superior to pegylated IFNα-2a alone (52), suggesting that the pegylation might interfere with the interaction between Tα1 and IFN. The combination therapy with IFNα and Tα1 was also evaluated in chronic hepatitis C and proved effective (53), despite earlier reports indicating that Tα1 as single therapy did not show treatment benefits (54, 55). The efficacy of the combination therapy in chronic hepatitis C was confirmed in randomized studies (56–58), although the higher efficacy was achieved when end-of-treatment rather than sustained responses were evaluated. Collectively, these results support the hypothesis that a combination regimen in chronic hepatitis B and C may be of therapeutic efficacy, with Tα1 likely promoting the optimal conditions for the full exploitation of the biological effects of IFNα. This is supported by *in vitro* studies in which IFNα and Tα1 inhibited clonal growth of hepatitis B transfected HepG2 hepatoblastoma cells more efficiently than the single agents alone (59). Similarly, while IFNα and Tα1 alone did not significantly inhibit HBV-DNA production in the culture supernatant from HBV-HepG2 cells, the combination of Tα1 and IFNα resulted in a statistically significant inhibition of virus production (60). Along the same line, treatment of PBMCs from patients with chronic hepatitis C with Tα1 increased Th1 and decreased Th2 cytokines, an effect that was potentiated by combined treatment with Tα1 and IFNα, including the synthesis of the antiviral protein 2′,5′-oligoadenylate synthetase (61).

With the introduction of nucleoside analogs in the management of chronic hepatitis B, studies have been performed to evaluate the safety and efficacy of Tα1 in combination with nucleoside analogs. A study evaluating the combination of Tα1 and famciclovir demonstrated a greater reduction of HBV-DNA levels when compared to famciclovir alone and serological clearance of HBeAg was associated with activation of HBV-specific Th1 cells (62). Similarly, a meta-analysis based on eight trials (583 patients in total) showed that the lamivudine and Tα1 combination was significantly superior to lamivudine alone in terms of ALT normalization rate, virological response rate, and HBeAg seroconversion rate (63). Finally, the combination entecavir and Tα1 was more effective than entecavir alone in improving ALT, HBV-DNA, HBeAg, and HBeAg seroconversion (64), and, although not significantly, showed a tendency to inhibit the development of hepatocellular carcinoma (HCC) in patients with HBV-related compensated cirrhosis (65). In the treatment of chronic hepatitis C, the addition of Tα1 to IFNα, pegylated or not, and ribavirin in patients who have failed prior interferon and ribavirin treatment (66–69) has shown promising results. These observations led to a phase III trial on 552 patients to determine whether addition of Tα1 to the standard of care Peg-IFNα-2a and ribavirin in non-responders could improve the response rates (70). The results of the study, however, indicated that Tα1 did not increase the on-treatment viral response, but, as discussed by the authors, were suggestive of an adjuvant role of Tα1 to prevent relapses in patients that achieved a virological response during therapy (70).

Collectively, the experience with Tα1 in chronic hepatitis B and C would suggest a clinical benefit when used in combination with antiviral agents to provide a delayed protection by sustaining a proper immune response. The associated favorable safety profile is an obvious advantage and might help to prevent evolution of chronic hepatitis into hepatocellular carcinoma.

### Thymosin α1 as a Therapy of HCC

Besides its use in the prevention of HCC in chronic hepatitis B and C, Tα1 has also been used in therapeutic treatment of HCC. A recent report has retrospectively evaluated the use of Tα1 as adjuvant therapy in patients with primary HBV-related small HCC after liver resection. As compared to patients that received only liver resection, patients treated with Tα1 had higher overall survival and recurrence-free survival, together with a reduced neutrophil-to-lymphocyte ratio, pointing to the use of Tα1 in patients at high risk for recurrence after resection (71). The administration of Tα1 also proved effective in improving liver function and increasing overall survival and recurrence-free survival in a retrospective study evaluating patients with HBV-associated HCC after radical hepatectomy (72).

Several studies have been performed to assess the use of Tα1 in unresectable HCC. Patients who are not candidate for surgery, but have tumors small enough for ablative therapy, transarterial chemoembolization (TACE) may prolong survival and addition of Tα1, along with an excellent safety profile, may improve outcomes (73, 74). The addition of Tα1 to TACE (75) or lamivudine (76) may also be useful in post-operative treatment to prevent recurrence. Finally, in advanced hepatocellular carcinoma, the addition of Tα1 to the kinase inhibitor sorafenib increased the median survival time and immune parameters (77). Collectively, these results indicate that Tα1 might be used not only to prevent development of HCC from chronic hepatitis, but also to treat HCC once established, either the resectable and unresectable forms, most likely by keeping HBV replication and recurrences under control.

## Thymosin α1 and Lung Cancer

Following the early clinical studies on Tα1 in lung cancer patients (78, 79), showing that Tα1 treatment was associated with significant improvements in immune parameters, and prolonged relapse-free and overall survival, especially for patients with non-bulky tumors, subsequent studies have focused on the combination of Tα1 with chemotherapy in mouse models and human patients. In parallel with their study in melanoma bearing mice described above, Pica et al. first demonstrated that the combination of Tα1 with αβ-IFN restored NK cell activity in mice inoculated with LLC cells (17) and that prolonged combined treatment with Tα1 and αβ-IFN or IL-2 significantly reduced tumor growth (19). Then, the authors showed that if the Tα1 and αβ-IFN treatment was preceded by Cy, it was possible to eradicate the tumor in LLC bearing mice, and this was associated with enhanced NK cell activity and long-term survival (80). Similarly, the combination of Tα1 and IL-2 after Cy treatment induced complete tumor regression (24), thus reinforcing the notion that combining immunotherapy with chemotherapy might be an effective anti-tumor strategy.

Based on these premises, clinical studies were performed incorporating the combination of Tα1 and IFN in the non-small cell lung cancer anti-tumor regimen. Garaci et al. first assessed a sequential chemoimmunotherapy protocol based on cisplatin, etoposide, Tα1, and IFN-α2a (81). Objective response was observed in 24 out of 56 patients, with a median survival of 12.3 months, and improved NK cells activity and absolute CD4 and CD8 T cell numbers (81). Along the same line, the treatment with Tα1 and low-dose IFNα after ifosfamide resulted in a trend toward enhanced response rate and a significant difference in time to progression (82). This was associated with normalized CD4+, CD8+, and NK cell counts (82), thus confirming the immune stimulating effect of Tα1 and low doses of IFNα. In addition, a meta-analysis on 10 randomized controlled trials including 724 patients evaluating Tα1 in combination with cisplatin and vinorelbine or gemcitabine, could show that the addition of Tα1 increased overall response rate, tumor control rate, CD4+ and NK cells (83), again supporting the notion that Tα1, by restoring the immune capabilities, might adjuvate standard chemotherapy for improved anti-tumor effects. While the effects of Tα1 on immune effector cells are evident from these studies, it is noteworthy that Tα1 can also activate immunosuppressive cells with untoward effects in the anti-tumor response. Indeed, by using a LLC model it was shown that, while the combination of Tα1 and gemcitabine could reduce tumor growth more effectively that gemcitabine alone, the only treatment with Tα1 was ineffective (84). Indeed, although Tα1 alone was able to induce CD8+ cells, similar to the combination with gemcitabine, it also activated myeloid-derived suppressor cells by upregulating arginase 1, thus impairing the anti-tumor activity (84). These results highlight the concept that Tα1 has pleiotropic effects and redirecting Tα1 activities by combination with other drugs might be crucial to observe the desired effects.

Besides its action on immune cells, Tα1 can also directly impacts tumor cells. Indeed, Moody and coworkers demonstrated that Tα1 could bind to the surface of human non-small cell lung cancer cells and inhibit their proliferation *in vitro* and xenograft formation in nude mice (85). The same authors also demonstrated that Tα1 could prevent lung adenomas in A/J mice injected with carcinogens, such as urethane, with Tα1 being more efficient in the early phase, when lung adenomas were small (86, 87). This was paralleled by the ability of Tα1 to directly inhibit the proliferation of non-tumorigenic and tumorigenic mouse lung cells (87). In addition, Tα1 was shown to inhibit not only the proliferation, but also the migration of the human lung epithelial adenocarcinoma cell line A549 (88). The effect on migration, however, was not confirmed in another study, in which it was shown that Tα1 significantly suppressed both *in vitro* and *in vivo* cell migration and invasion of certain NSCLC cells, but not others, including A549, the discriminant being the level of PD-L1 expression (89). Indeed, knock-down of PD-L1 impaired the ability of Tα1 to inhibit cell migration and invasion of cells expressing high levels of PD-L1 (89). Finally, the anti-proliferative effects of Tα1 on lung cancer cells could be potentiated by fusion with an iRGD sequence (90), that enhanced its tumor penetrating ability, in turn translating in higher antitumor effects (34), similarly to what observed in melanoma cells. Collectively, these results indicate that Tα1, by either interfering with tumor cells or modulating immune cells, can be beneficial in lung carcinoma, especially when combined with chemotherapeutic drugs that shift the pleiotropic activities of Tα1 toward tumor regression.

## Novel Concepts in Cancer Immunotherapy Open new Opportunities for Tα1 Application

Cancer immunotherapy, defined as the fourth pillar of human cancer therapy, next to surgery, chemotherapy and radiotherapy, has been employed for more than a century, starting from the experiments of Coley in 1890 where bacterial products proved beneficial for inoperable cancer (91), but only recently has gained a central place in cancer therapy (92). Distinct forms of cancer immunotherapies have been developed in the last three decades (93), and novel concepts have emerged that will be discussed in the context of checkpoint inhibitors.

Monoclonal antibodies targeting the immune checkpoints CTLA-4 and PD-1/PD-L1 have represented a breakthrough in the recent years for their ability to restore antitumor immunity, and have rapidly entered the clinical practice for the therapy of a variety of tumors (94). Despite undisputable success, a broad application in clinical practice is still limited by two major shortcomings. First, the efficacy is limited as the majority of tumor patients do not respond to the therapy, a phenomenon that has been linked to the tumor characteristics of immune infiltration (95). Specifically, tumors in which lymphocyte infiltration occurs (“hot” tumors) are more likely to respond to T cell checkpoint inhibition than tumors without lymphocyte infiltration (“cold” tumors) (95). Second, the safety is compromised by the emergence of immune-related adverse events that result from off-target effects of an excessively activated immune system (96). Collectively, this novel scenario delineates the framework for future work in cancer immunotherapy that encompasses two major directions: first, the tumor-immune microenvironment might be turned into a favorable configuration for immune checkpoint inhibitors to work; second, a parallel therapy should be envisaged to limit off-targets autoimmune effects.

**Figure 1:**
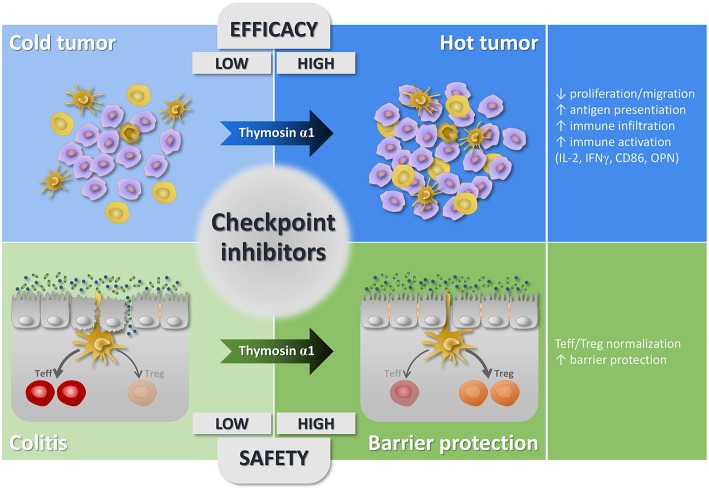
Schematic depiction of the potential application of Tα1 in combination with checkpoint inhibitors. The panel shows that checkpoint inhibitors might display low efficacy in the immune excluded, cold tumors and, at the same time, be associated with a low safety by impairing mucosal barrier integrity, for instance in the gastrointestinal tract. The panel also shows that the concomitant treatment with Tα1 might improve both the efficacy and safety of checkpoint inhibitors by turning a cold into a hot tumor and restoring mucosal homeostasis. Details are described in the text.

Based on this framework, several applications of Tα1 can be thought of that intersect these directions (Figure 1). Turning a cold into a hot tumor requires a priming therapy that enhances T cells responses and the concomitant removal of co-inhibitory signals and/or the supply of stimulatory signals (95). In addition to the T cell priming ability reported in the previous sections, Tα1 is endowed with additional properties that are very interesting at this purpose. First of all, Tα1 can induce MHC class I expression in tumor cells (97), thus increases the possibility of making tumor cells visible to T lymphocytes. Second, Tα1 fused with the Fc domain of human IgG4 plays anti-tumor effects in the 4T1 and B16.F10 tumor xenograft models by upregulating CD86 expression, secreting IFNγ and IL-2, and increasing the number of tumor-infiltrating CD4+ and CD8+ T cells (35). It is worth mentioning that low levels of IFNγ are characteristic of infiltration-excluded tumors (98), and elevating IFNγ may result in increased MHC class I and immunoproteasome expression for enhanced antigen presentation (95). Similarly, fusion of Tα1 with the tumor homing peptide iRGD results in increased T cell activation and CD86 expression in melanoma and lung cancer (34). Third, Tα1 directly targets NSCLC cells highly expressing PD-L1 to block proliferation and migration and might work cooperatively with an anti-PD-L1 antibody to enhance the immune response against the tumor (89). Fourth, the favorable combination of Tα1 with an anti-PD-1 antibody has been already postulated in an experimental setting in which low doses of Tα1, while being ineffective alone, increased the efficacy of an anti-PD-1 antibody in the lung metastasis melanoma model (30). Collectively, this experimental evidence strongly suggests that Tα1, either in the native form or modified to increase its half-life or tumor-homing properties, is a promising molecule to modify the tumor and its microenvironment and create the optimal conditions for the activity of immune checkpoint inhibitors.

Another potential field for Tα1 application is represented by the prevention of the adverse effects of immune-checkpoint inhibitors (Figure 1). Diarrhea and/or colitis are common adverse events associated with immune checkpoint inhibitors (99). It has been postulated that the immune enterocolitis caused by checkpoint inhibition is secondary to hyperactivated effector T cells targeting luminal antigens (that is, those from the microbiota and dietary products) and to loss of functional Treg cells (99). Interestingly, we have previously shown that Tα1 expands plasmacytoid Dendritic Cells (pDC) in bone marrow precursor cells stimulated with GM-CSF and IL-4 in a TLR9- and type I IFNR signaling-dependent manner (100). This population of pDC expressed indoleamine 2,3-dioxygenase and was necessary and sufficient to mediate antimicrobial immunity and alloantigen tolerization in experimental hematopoietic stem cell transplantation (100). This activity of pDCs involved the promotion of T helper type 1 immunity within a regulatory environment mediated by the induction of Treg cells (100). These results suggest that Tα1 may contribute to the induction and maintenance of peripheral tolerance and might play a role in promoting gastrointestinal homeostasis by normalizing the Teff/Treg ratio. Indeed, we have already shown that Tα1 can rescue IDO1 expression, tissue architecture, barrier function and cytokine balance in the small intestine of a murine model of cystic fibrosis, with a predominantly intestinal phenotype (101), and we have extended these results to show that Tα1 is also protective in other models of intestinal damage (M. M. B., personal communication). Interestingly, preliminary results indicate that Tα1 also protects against gut immune damage in mice with DSS-induced colitis exacerbated with anti-CTLA-4 and/or anti-PD-1 antibodies, one of the models currently employed to evaluate the toxicity of immune checkpoint inhibitors (102, 103). All in all, these results indicate that Tα1 may not only modulate the tumor immune environment for optimal efficacy of immune checkpoint inhibitors, but also normalize mucosal immunity for prevention of collateral damage. This activity of Tα1 on mucosal homeostasis may have further implications. For instance, it has been demonstrated that the microbiota, i.e. the microorganisms that colonize the human body, can modulate the efficacy of checkpoint inhibitors, and perturbations of its composition, for instance following administration of antibiotics, may have a significant negative impact (104). By promoting immune tolerance in the gut, Tα1 may sustain the integrity of the microbiome that may reflect in a higher efficacy of immune checkpoint blockade.

Lastly, in addition to polarize a subset of GM-CSF/IL-4 differentiated bone marrow precursors, Tα1 can also drive the development of DCs from bone marrow precursors by itself with peculiar morphological, phenotypical and functional characteristics, able to protect against *Aspergillus* infection in adoptive transfer experiments (M. M. B. et al., manuscript in preparation). More importantly, in the context of the present review, Tα1 bone marrow cultures up-regulated osteopontin (OPN) during differentiation and upon stimulation with microbial agonists. OPN has been the subject of intense research in cancer (105), and a wealth of evidence has been provided to demonstrate that OPN, secreted by tumor cells as well as infiltrating immune cells, promotes tumor growth and metastasis by supporting a pro-tumorigenic environment (105). However, OPN is present in both secreted and intracellular forms, and the functions may be different (106). For instance, intracellular OPN was found to inhibit TLR signaling in macrophages and ameliorate inflammatory pathology in diethylnitrosamine-induced hepatocarcinogenesis (107). In addition, the ratio between secreted and intracellular OPN may also be of relevance, as it can skew the balance between myeloid and lymphoid populations in pathogenic conditions, such as infection and autoimmunity (108). Since an expansion of immature or dysfunctional myeloid cells and a decline in the quantity and quality of the lymphoid response is observed in tumors (109), it is tempting to speculate that the ratio between secreted and intracellular OPN may be affected in the tumorigenic process and potentially targeted for therapeutic intervention. Therefore, the ability of Tα1 to upregulate OPN during DC differentiation is certainly of relevance in tumor immunology, but should be carefully directed to polarize the pleiotropic effects of OPN toward an anti-tumorigenic effect.

## Conclusions

More than four decades have now elapsed from the isolation of Tα1 and the original observations on its immunostimulatory activities have been confirmed, enriched and extended by several studies in both animal models and human patients. From the beginning, one of the major interests for the potential application of Tα1 has been represented by tumors, in the belief that Tα1 could restore or potentiate an immune system that was suppressed by the tumor itself and the concomitant therapies. And Tα1 did not fall short of expectations. Indeed, promising results, more often when used in combination with other therapies, were obtained in different types of tumors. Apparently, however, the research on Tα1 did not keep up with the recent exciting developments of cancer immunotherapy, profoundly marked by the clinical application of checkpoint inhibitors, and the potential use of Tα1 required to be revisited under a new perspective. More recent results on the immunomodulatory effects of Tα1, combined with increased knowledge of host/tumor response to checkpoint inhibitors, has shown the way to future research with Tα1 in cancer therapy that should include, holistically, the response of the tumor, the tumor microenvironment, and the distant sites. Indeed, turning a cold into a hot tumor and promoting immune cell infiltration to increase the efficacy of checkpoint inhibitors, and potentiating the mucosal barrier at distant sites to limit side effects, are all within range of Tα1 activity and promising developments are expected in the next future.

## Author Contributions

CC and LR wrote the paper with substantial contributions from all authors listed.

### Conflict of Interest Statement

The authors declare that the research was conducted in the absence of any commercial or financial relationships that could be construed as a potential conflict of interest.
